# Effects of maltreatment and parental schizophrenia spectrum disorders on early childhood social-emotional functioning: a population record linkage study

**DOI:** 10.1017/S204579601600055X

**Published:** 2016-08-04

**Authors:** S. L. Matheson, M. Kariuki, M. J. Green, K. Dean, F. Harris, S. Tzoumakis, M. Tarren-Sweeney, S. Brinkman, M. Chilvers, T. Sprague, V. J. Carr, K. R. Laurens

**Affiliations:** 1 School of Psychiatry, University of New South Wales, Sydney, Australia; 2 Schizophrenia Research Institute, Sydney, Australia; 3 Neuroscience Research Australia, Sydney, Australia; 4 Justice Health and Forensic Mental Health Network, Sydney, Australia; 5 School of Health Sciences, Canterbury University, Christchurch, New Zealand; 6 School of Medicine and Public Health, Newcastle University, Newcastle, Australia; 7 Telethon Kids Institute, The University of Western Australia, Perth, Australia; 8 School of Population Health, The University of Adelaide, Perth, Australia; 9 New South Wales Department of Family and Community Services, Sydney, Australia; 10 New South Wales Ministry of Health, Sydney, Australia; 11 Department of Psychiatry, Monash University, Melbourne, Australia

**Keywords:** Behaviour problems, child abuse, neglect, mental health, risk factors

## Abstract

**Aims.:**

Childhood maltreatment and a family history of a schizophrenia spectrum disorder (SSD) are each associated with social-emotional dysfunction in childhood. Both are also strong risk factors for adult SSDs, and social-emotional dysfunction in childhood may be an antecedent of these disorders. We used data from a large Australian population cohort to determine the independent and moderating effects of maltreatment and parental SSDs on early childhood social-emotional functioning.

**Methods.:**

The New South Wales Child Development Study combines intergenerational multi-agency data using record linkage methods. Multiple measures of social-emotional functioning (social competency, prosocial/helping behaviour, anxious/fearful behaviour; aggressive behaviour, and hyperactivity/inattention) on 69 116 kindergarten children (age ~5 years) were linked with government records of child maltreatment and parental presentations to health services for SSD. Multivariable analyses investigated the association between maltreatment and social-emotional functioning, adjusting for demographic variables and parental SSD history, in the population sample and in sub-cohorts exposed and not exposed to parental SSD history. We also examined the association of parental SSD history and social-emotional functioning, adjusting for demographic variables and maltreatment.

**Results.:**

Medium-sized associations were identified between maltreatment and poor social competency, aggressive behaviour and hyperactivity/inattention; small associations were revealed between maltreatment and poor prosocial/helping and anxious/fearful behaviours. These associations did not differ greatly when adjusted for parental SSD, and were greater in magnitude among children with *no history* of parental SSD. Small associations between parental SSD and poor social-emotional functioning remained after adjusting for demographic variables and maltreatment.

**Conclusions.:**

Childhood maltreatment and history of parental SSD are associated independently with poor early childhood social-emotional functioning, with the impact of exposure to maltreatment on social-emotional functioning in early childhood of greater magnitude than that observed for parental SSDs. The impact of maltreatment was reduced in the context of parental SSDs. The influence of parental SSDs on later outcomes of maltreated children may become more apparent during adolescence and young adulthood when overt symptoms of SSD are likely to emerge. Early intervention to strengthen childhood social-emotional functioning might mitigate the impact of maltreatment, and potentially also avert future psychopathology.

## Introduction

Childhood social-emotional dysfunction is a potentially modifiable antecedent that precedes the development of schizophrenia spectrum disorders (SSDs) (Tarbox & Pogue-Geile, 2008; Welham *et al*. 2009; Matheson *et al*. 2013*a*; Laurens *et al*. 2015). Birth cohort studies demonstrate that, when aged 4–7 years, children who later develop SSDs are more likely to engage in solitary play (Jones *et al*. 1994), show social maladjustment (Bearden *et al*. 2000), and suffer peer rejection, and internalising and externalising problems (Cannon *et al*. 2002). Whether these problems are partially accounted for by established risk factors for SSD, namely prior exposure to social stressors such as childhood maltreatment, and/or by familial risk for SSDs, has not been examined sufficiently.

Animal studies indicate that sustained exposure to stress has long-term effects on social withdrawal, aggression and anxiety (Sandi & Haller, 2015). Childhood maltreatment has been associated with poor social-emotional functioning in small, case-control studies of 3–8 year-olds exposed to early maltreatment (Anthonysamy & Zimmer-Gembeck, 2007; Milot *et al*. 2010). Such associations appear to be present irrespective of type of maltreatment experienced. Physical maltreatment has been associated with heightened aggression and hyperactivity in 5–8 year-olds (Prino & Peyrot, 1994), and in 8–12 year-olds who also show low peer status and poor cooperation (Salzinger *et al*. 1993). Sexual (Tyler, 2002) and emotional maltreatment (Schneider *et al*. 2005; Shaffer *et al*. 2009) have each been associated with both internalising and externalising behaviours in children aged 3–8 years, while neglect has been associated with social withdrawal in pre-schoolers (Hildyard & Wolfe, 2002), and in 5–8 year-olds (Prino & Peyrot, 1994). Differential effects of maltreatment have been reported in 4–12 year-olds according to sex, with internalising and externalising behaviours increasing in females over time but decreasing in males over time, though sex differences in maltreatment effects in this age group are not identified consistently (Vachon *et al*. 2015).

Meta-analyses identify a three-fold increased risk of subsequent adult SSDs in children exposed to maltreatment (Varese *et al*. 2012; Matheson *et al*. 2013*b*). Recent extensions of the neurodevelopmental hypothesis of schizophrenia postulate that exposure to ongoing stress in children may promote and interact with behavioural problems in the genesis of adult SSDs, particularly in the presence of genetic vulnerability for these disorders (Morgan *et al*. 2010; Howes & Murray, 2014). Having a parent with a SSD is a proxy indicator of genetic vulnerability to these disorders, with risk increasing from a ~1% in the general population to 13% if one parent has schizophrenia (Gottesman & Erlenmeyer-Kimling, 2001). Parental SSDs are associated directly with early social-emotional dysfunction in offspring (Hanson *et al*. 1976; Niemi *et al*. 2003; Henriksson & McNeil, 2004).

It is difficult to disentangle the effects of inherited vulnerability for SSDs from maltreatment on both early social-emotional dysfunction and adult psychosis. A small study of children (*n* = 144; mean age ~10 years, and followed for 1 year) identified generalised effects of childhood maltreatment on aggression, delinquency and social withdrawal, but found no interaction of maltreatment with parental schizophrenia, though the analysis may have lacked power (Bergman & Walker, 1995). Similarly, exposure to childhood maltreatment in the context of a family history of psychosis conferred no greater likelihood of later psychosis than the estimated risk among maltreated individuals without a family history of psychosis, suggesting that the effects of maltreatment are independent of parental history of disorder (Fisher *et al*. 2014).

In the present study, we used a large population cohort of children to investigate the association between maltreatment and multiple indices of early childhood social-emotional functioning, with consideration of the effects of parental SSDs on this functioning. We hypothesised that children exposed to maltreatment would show greater social-emotional dysfunction than children not exposed to maltreatment, and that this relationship would remain after adjusting for history of parental SSDs. We further sought to confirm that the relationship between maltreatment and social-emotional functioning held both for children exposed and not exposed to parental SSDs. We anticipated also that children exposed to parental SSDs would show greater social-emotional dysfunction than children without a history of parental SSDs, and that the relationship would hold after adjusting for exposure to maltreatment. For both childhood maltreatment and parental SSD exposures, we expected pervasive effects across a range of social-emotional functioning outcomes.

## Method

### Study design and sample

The New South Wales Child Development Study (NSW-CDS) is an Australian longitudinal population-based cohort study designed to identify childhood risk and protective factors for a variety of mental health and social outcomes in childhood, adolescence and adulthood (Carr *et al*. 2016). It utilises intergenerational multi-agency record linkage to combine data for 87 026 children and their parents. This study used linked data that provided information on exposures and outcomes during the early childhood period (birth to 5 years). Ethical approval was obtained from the NSW Population and Health Services Research Ethics Committee (HREC/11/CIPHS/14), with associated data custodian approvals granted by the relevant Government Departments.

The NSW-CDS cohort was defined in 2009 when teachers in government and private schools nation-wide completed the Australian Early Development Census (Brinkman *et al*. 2007; Australian Government, 2009; Brinkman *et al*. 2014), a validated measure of child development in multiple domains [the Canadian Early Development Index (EDI) used in Australia was modified by excluding 9 items and originally named the ‘Australian Early Development Index’ (AEDI); it was later renamed to the ‘Australian Early Development Census’ (AEDC)]. The AEDC was completed during the children's first year of full-time formal schooling (kindergarten) – at around 5 years of age – by teachers with a minimum of 1 month's knowledge of the child. The NSW-CDS cohort captured 99.7% of NSW children enrolled in kindergarten in 2009, and is representative of the Australian population of comparable age (Carr *et al*. 2016).

Linkage of child AEDC records with a variety of data collections is described elsewhere (Carr *et al*. 2016). This study utilised child protection and parental mental health information available from the NSW Department of Family and Community Services (FACS) *Case Management System* (*Key Information Directory System*) (*CMS*[*KiDS*]), and the NSW Ministry of Health *Mental Health Ambulatory Data Collection* and *Admitted Patients Data Collection* respectively. Intergenerational linkage of child and parent data was conducted using birth registration records available in the *NSW Register of Births, Deaths and Marriages – Birth Registrations*; linked parent data were available only for children whose births were registered in NSW. From the NSW-CDS cohort, records from 14 781 children born outside of NSW (for whom no parent linkage could be conducted) and from 3129 children lacking AEDC social-emotional function ratings due to the presence of special needs were excluded. The final sample therefore comprised 69 116 children. Data on social-emotional functioning in this sub-cohort was comparable with that reported for the full NSW-CDS cohort (Carr *et al*. 2016).

### Social-emotional functioning outcomes

Vulnerability scores on five subdomains of social-emotional functioning assessed by the AEDC (Australian Government, 2009) measured: (i) poor social competence (e.g., inability to get along with peers), (ii) poor pro-social and helping behaviour (e.g., unwillingness to help others in need), (iii) anxious and fearful behaviour (e.g., worrying, nervousness), (iv) aggressive behaviour (e.g., physical aggression, bullying) and (v) hyperactive and inattentive behaviour (e.g., distractibility, impulsivity). Categorisation as ‘developmentally vulnerable’ on each subdomain was determined by a score in the bottom 10% of the national 2009 AEDC (Brinkman *et al*. 2014).

### Childhood maltreatment

Exposure to maltreatment was examined in three ways:

*Any maltreatment:* Exposure to any childhood maltreatment was determined using *CMS*[*KiDS*] child protection reports, where a designation of ‘actual harm’ or ‘risk of significant harm’ had been indicated by a FACS case worker, following case review, to determine that the child had been, was being, or was likely to be abused, neglected, or otherwise harmed. Exposure to actual harm or risk of significant harm was coded using a dichotomous variable indicating the presence of ‘any maltreatment’ *v*. ‘no maltreatment’. Children with a *CMS*[*KiDS*] report *prior to* the AEDC assessment were treated as having been exposed to maltreatment, while children with a report *only after* the AEDC assessment were regarded as not exposed.

*Maltreatment types:* Four different types of maltreatment were identified by the FACS case worker completing a report as the primary type of maltreatment on each referral occasion: (i) physical maltreatment (i.e., being physically assaulted, kicked, hit, or bitten), (ii) emotional maltreatment (i.e., being insulted, unjustly punished or treated, threatened, or belittled), (iii) sexual maltreatment (i.e., indecent acts, molestation, or penetration) and (iv) neglect (i.e., being abandoned or receiving inadequate care). Each type of maltreatment was coded dichotomously as present *v*. absent.

*Diversity of maltreatment:* This variable coded the number of different types of maltreatment reported for a given child over the period of observation (birth until AEDC assessment), namely: exposure to no maltreatment, one type of maltreatment, or two or more types of maltreatment. Exposure to multiple types of maltreatment has been robustly related to poor developmental outcomes in young children (Lau *et al*. 2005). Moreover, children exposed to multiple types of maltreatment tend to be chronically exposed (English *et al*. 2005).

### Parental history of SSDs

Parental SSDs were determined by identifying parents of the child cohort who had relevant mental health records within the NSW Ministry of Health's *Mental Health Ambulatory Data Collection* and *Admitted Patients Data Collection*, using methods developed previously (Sara *et al*. 2014). We included the following ICD 10 diagnoses: schizophrenia, schizoaffective disorder, other non-affective psychotic disorders and cluster A personality disorders (schizotypal disorder, paranoid personality disorder and schizotypal personality disorder). Admitted patients’ diagnoses were recorded at the time of discharge from public or private hospitals, based on assessment by the treating psychiatrist. Ambulatory (outpatient) diagnoses were recorded by treating clinicians at each community contact. As different frequencies of service contact characterised these data collections, diagnostic data from multiple presentations in the ambulatory collection were reduced to more closely equate to the single diagnosis given on discharge from hospital. *Mental Health Ambulator*y data were grouped into 3-month (i.e., quarterly) periods, and the ‘last specific’ diagnosis within each quarter was ascribed as the diagnosis for that period (Sara *et al*. 2014). Inpatient and outpatient episodes of care were then combined, and the occurrence of *any* diagnosis of parental SSD in these records of care was used to designate children as exposed to parental SSD. Some children had parents with multiple SSD diagnoses within their care records (e.g., some parents had received both schizophrenia and schizoaffective disorder diagnoses during separate [repeat] presentations to health services). This exposure variable was coded dichotomously as presence *v*. absence of parental SSD.

### Age, sex and socio-economic status (SES)

Age, sex and SES for each child were obtained from the AEDC and entered into the analyses as covariates due to their potential confounding effect on the association between maltreatment and social-emotional functioning (Niemi *et al*. 2005; Thompson & Tabone, 2010). SES was indexed by the Socio-Economic Index for Areas [SEIFA: (Australian Government, 2011), applied to children on the basis of their suburb of primary home residence using Australian Bureau of Statistics data. SEIFA quintile scores span five levels from the most (SEIFA 1) to least disadvantaged (SEIFA 5). For analyses, these were grouped dichotomously into most disadvantaged (SEIFA levels 1 and 2) and least disadvantaged (SEIFA levels 3, 4 and 5).

As some previous research has identified differential effects of maltreatment on social-emotional functioning in young females and males (Godinet *et al*. 2014), and because vulnerability rates on the AEDC subdomains vary by sex (Brinkman *et al*. 2012), we provide results of analyses conducted separately by sex in supplementary material.

### Data analysis

Data analysis was conducted using SAS software, version 9.4 (Proglang, 2013). Odds ratios (ORs) and their 95% confidence intervals (CIs) were calculated in bivariate and multivariable logistic regressions. Effect sizes were determined as ORs, with 1.00–2.00 (or 1.00–0.50) interpreted as small, 2.00–5.00 (or 0.50–0.20) interpreted as medium, and >5 (or <0.20) interpreted as large (Rosenthal, 1996). Statistical significance was reached when CIs did not cross 1.00.

#### Bivariate analyses

These analyses were conducted to determine the associations between social-emotional functioning and exposure variables, unadjusted for covariates; that is, associations between each social-emotional subdomain vulnerability score and: (a) any maltreatment (relative to no maltreatment); (b) the four maltreatment types (physical, sexual, emotional, or neglect; each type compared separately with no maltreatment); and (c) diversity of maltreatment (exposure to one type, or two or more types of maltreatment, relative to no maltreatment). Bivariate analyses also examined the relationships between each social-emotional subdomain vulnerability score and parental history of SSDs (present/absent).

#### Multivariable regressions

A series of logistic regression analyses were conducted to examine the association between the maltreatment exposures and the social-emotional outcomes following adjustment for covariates. Age, sex and SES were entered into the analysis first, and then the analysis was repeated with the addition of the parental SSD variable. Similarly, to assess the independent effects of any maltreatment on the association between parental SSDs and social-emotional functioning, the analysis was conducted firstly with adjustment for age, sex and SES, and then repeated with the addition of exposure to any maltreatment. Finally, to examine whether the associations were similar in children with and without parental history of SSD, we present the associations between exposure to any maltreatment and social-emotional functioning (both unadjusted, and following adjustment for age, sex and SES), stratified by the presence *v*. absence of parental SSDs. The study was underpowered to undertake a formal assessment of statistical interaction between maltreatment and parental SSD (using the AEDC Social Competence domain as an example, in our sample of 69 116 children we had power of only 0.09 to detect an interaction effect of small magnitude [OR = 1.5] and power of 0.65 to detect an interaction effect of large magnitude [OR = 5.0]).

## Results

### Sample characteristics

Demographic characteristics of the sample for analysis (*N* = 69 116 children) are provided in Table 1, along with the prevalence of maltreatment and parental SSD exposures. Data on the prevalence of maltreatment in our sample (2.9%) spanned a 6-year period (i.e., the years 2003–2009; incorporating birth to 5 years of age). Recent estimates indicate a 12-month prevalence of maltreatment Australia-wide among children aged 0–17 years of 2.7% (Australian Institute of Health and Welfare, 2015), and rates for maltreatment occurring before age 18 years of 2.4% (for sexual abuse), 5.3% (for physical abuse), and 4.4% (for neglect) in other high-income countries [Belgium, France, Germany, Israel, Italy, Japan, The Netherlands, Spain, USA; (Kessler *et al*. 2010)]. The prevalence of parental SSD (1.2%) in our sample derived from data spanning a decade prior to the AEDC assessment (i.e., the years 2000–2009). A recent estimate based on more broadly-defined psychotic disorders (i.e., also including affective psychoses) in Australia for 12-month treated prevalence (in public services) was 0.45% (Morgan *et al*. 2012); and a recent worldwide lifetime prevalence estimate for schizophrenia only based on 29 studies was 0.48% [interquartile range: 0.34–0.85%; (Simeone *et al*. 2015)].

**Table 1.** Sample characteristics (N = 69 116 children)

	Mean	(s.d.)
Age (years)	5.6	(0.4)
	*N*	(%)
Sex (female)	34 185	(49.5)
Rated disadvantaged on SEIFA	31 076	(45.0)
Children with child protection reports	2041	(2.9)
Maltreatment types^a^		
Physical	460	(0.6)
Emotional	1122	(1.6)
Sexual	223	(0.3)
Neglect	721	(1.0)
Diversity of maltreatment		
One type	1626	(2.3)
Two or more types	415	(0.6)
Parental SSD^b^	835	(1.2)
Both parental SSD and child protection report^c^	237	(0.3)

*Note*: s.d., standard deviation; SEIFA, Socio-Economic Index for Areas; SSD, schizophrenia spectrum disorder.

a For every maltreatment type, greater than half of children had solely a report (or multiple reports) of that type (physical: 261 children; emotional: 778; sexual: 154; neglect: 433).

b Of the 835 children with parental SSD, 828 had a parent with a diagnosis of schizophrenia, schizoaffective disorder and/or other non-affective psychotic disorders (the remaining seven children had a parent with a diagnosis of a cluster A personality disorder only).

c Representing 12.0% of children with a child protection record, and 28.4% of children with parental SSD history.

### Association of maltreatment and social-emotional functioning

Table 2 presents the results of unadjusted and adjusted analyses examining the association between exposure to any maltreatment and the five social-emotional outcomes. There were small attenuations of the ORs after adjusting for age, sex and SES, and then further small attenuations when adjusting for parental SSD. The fully adjusted associations between any maltreatment and poor social competency, aggressive behaviour and hyperactivity/inattention were of medium magnitude. Associations between any maltreatment exposure and poor prosocial/helping and anxious/fearful behaviour were small. The ORs observed for females were slightly larger in magnitude than those for males across all subdomains, though the overlap in confidence intervals indicated a lack of significant differences in effect across sexes (see supplementary tables).

**Table 2.** Associations between any maltreatment and socio-emotional functioning

	Poor social competency		Poor prosocial/helping behaviour		Anxious/fearful behaviour		Aggressive behaviour		Hyperactivity/inattention	
	OR (95% CI)	*N*	OR (95% CI)	*n*	OR (95% CI)	*n*	OR (95% CI)	*n*	OR (95% CI)	*N*
		324		245		380		443		498
U	3.5 (3.1–4.0)		1.8 (1.6–2.1)		2.0 (1.8–2.3)		3.3 (3.0–3.7)		2.9 (2.6–3.3)	
A_1_	3.3 (2.9–3.7)		1.8 (1.5–2.0)		1.9 (1.7–2.2)		3.3 (2.9–3.6)		3.0 (2.7–3.3)	
A_2_	3.1 (2.7–3.5)		1.7 (1.5–2.0)		1.8 (1.6–2.1)		3.2 (2.8–3.5)		2.8 (2.5–3.1)	

*Note*: The reference group for each analysis is children experiencing no maltreatment; *n* = number of developmentally vulnerable children with maltreatment exposure; OR, odds ratio; CI, confidence intervals; U, unadjusted; A_1_, adjusted for age, sex and socio-economic status; A_2_, adjusted for age, sex, socio-economic status and parental schizophrenia spectrum disorder.

Unadjusted and adjusted associations between each of the four types of maltreatment and the five social-emotional outcomes are presented in Table 3a, with little attenuation of the ORs observed following adjustment for demographic covariates and then parental SSDs. In the fully adjusted analyses, each type of maltreatment showed medium-sized effects on poor social competency, aggressive behaviour, and hyperactive-inattentive behaviour, and small- to medium-sized effects on prosocial/helping behaviour and anxious/fearful behaviour. Table 3b presents the results of unadjusted and adjusted analyses of the associations between the diversity of maltreatment exposure and social-emotional functioning. As before, adjustment for demographic covariates and then parental SSDs effected a small attenuation in the ORs. For poor social competency, anxious/fearful behaviour, aggressive behaviour and hyperactive-inattentive subdomains, dose-dependent associations between exposure to one type of maltreatment and two or more types of maltreatment were apparent in the fully adjusted models (increasing to a large magnitude of effect between two or more types of maltreatment and poor social competency and aggressive behaviour). For prosocial/helping behaviour, the effect sizes also increased in magnitude, though not to the same extent.

**Table 3.** Associations between (a) each type of maltreatment and (b) diversity of maltreatment and socio-emotional functioning

	Poor social competency		Poor prosocial/helping behaviour		Anxious/fearful behaviour		Aggressive behaviour		Hyperactivity/inattention	
	OR (95% CI)	*n*	OR (95% CI)	*n*	OR (95% CI)	*N*	OR (95% CI)	*n*	OR (95% CI)	*n*
(*a*) *Type of maltreatment*										
Physical maltreatment		89		48		90		126		127
U	4.5 (3.6–5.7)		1.6 (1.2–2.1)		2.2 (1.8–2.8)		4.5 (3.7–5.6)		3.5 (2.9–4.3)	
A_1_	4.2 (3.3–5.3)		1.4 (1.1–2.0)		2.1 (1.7–2.6)		4.4 (3.6–5.5)		3.5 (2.8–4.3)	
A_2_	3.9 (3.1–5.0)		1.4 (1.0–1.9)		2.0 (1.6–2.5)		4.3 (3.4–5.3)		3.3 (2.7–4.1)	
Emotional maltreatment		198		132		220		256		293
U	4.0 (3.4–4.7)		1.8 (1.5–2.2)		2.1 (1.8–2.5)		3.4 (3.0–4.1)		3.2 (2.8–3.7)	
A_1_	3.6 (3.1–4.3)		1.7 (1.4–2.0)		2.0 (1.7–2.4)		3.4 (2.9–3.9)		3.2 (2.7–3.6)	
A_2_	3.3 (2.8–3.9)		1.6 (1.3–2.0)		1.9 (1.6–2.2)		3.2 (2.8–3.8)		2.9 (2.5–3.4)	
Sexual maltreatment		32		27		49		41		46
U	3.1 (2.1–4.5)		1.8 (1.2–2.7)		2.4 (1.8–3.3)		2.6 (1.9–3.7)		2.3 (1.7–3.2)	
A_1_	3.3 (2.3–4.9)		2.1 (1.4–3.1)		2.5 (1.8–3.4)		3.1 (2.2–4.4)		2.9 (2.1–4.1)	
A_2_	3.2 (2.2–4.6)		2.0 (1.3–3.1)		2.4 (1.7–3.3)		3.1 (2.2–4.3)		2.8 (2.0–4.0)	
Neglect		121		100		132		175		197
U	3.8 (3.1–4.6)		2.2 (1.7–2.7)		2.0 (1.6–2.4)		3.8 (3.2–4.5)		3.4 (2.9–4.0)	
A_1_	3.5 (2.9–4.3)		2.1 (1.7–2.6)		1.9 (1.5–2.3)		3.7 (3.1–4.5)		3.4 (2.9–4.1)	
A_2_	3.3 (2.7–4.0)		2.0 (1.6–2.5)		1.8 (1.5–2.2)		3.6 (3.0–4.3)		3.2 (2.7–3.9)	
(*b*) *Diversity of maltreatment*										
1 type		225		190		284		312		355
U	3.0 (2.6–3.5)		1.8 (1.5–2.1)		1.9 (1.6–2.1)		2.8 (2.5–3.2)		2.5 (2.2–2.9)	
A_1_	2.8 (2.4–3.3)		1.7 (1.5–2.0)		1.8 (1.6–2.0)		2.8 (2.4–3.2)		2.6 (2.3–2.9)	
A_2_	2.7 (2.3–3.1)		1.7 (1.4–2.0)		1.7 (1.5–1.9)		2.7 (2.4–3.1)		2.4 (2.1–2.7)	
**≥**2 types		99		55		96		131		143
U	5.9 (4.7–7.4)		2.0 (1.5–2.7)		2.6 (2.1–3.3)		5.5 (4.5–6.8)		4.8 (3.9–5.9)	
A_1_	5.4 (4.3–6.8)		1.9 (1.4–2.6)		2.5 (2.0–3.1)		5.4 (4.4–6.8)		4.9 (3.9–6.1)	
A_2_	5.0 (4.0–6.4)		1.9 (1.4–2.5)		2.4 (1.9–3.0)		5.3 (4.2–6.5)		4.6 (3.7–5.7)	

*Note*: The reference group for each analysis is children experiencing no maltreatment; *n* = number of developmentally vulnerable children with maltreatment exposure; OR, odds ratio; CI, confidence intervals; U, unadjusted; A_1_, adjusted for age, sex and socio-economic status; A_2_, adjusted for age, sex, socio-economic status and parental schizophrenia spectrum disorder.

### Association of parental SSDs and social-emotional functioning

Bivariate analysis indicated that having a parental history of SSD and a report of any maltreatment was strongly associated: OR = 14.6 (95% CI 12.5–17.1); 28% of children with a parental SSD had a maltreatment report. Results of unadjusted and adjusted analyses examining the association between parental SSDs and the five social-emotional subdomains are presented in Table 4. Adjusting for age, sex and SES had minimal effects on the associations, but a greater attenuation of the associations was apparent after also adjusting for any maltreatment, and effect sizes were all small following adjustment.

**Table 4.** Associations between parental schizophrenia spectrum disorder and socio-emotional functioning

	Poor social competency		Poor prosocial/helping behaviour		Anxious/fearful behaviour		Aggressive behaviour		Hyperactivity/inattention	
	OR (95% CI)	*n*	OR (95% CI)	*n*	OR (95% CI)	*n*	OR (95% CI)	*N*	OR (95% CI)	*N*
		105		86		149		126		175
U	2.6 (2.1–3.1)		1.3 (1.2–1.9)		1.9 (1.6–2.2)		2.0 (1.6–2.4)		2.3 (2.0–2.7)	
A_1_	2.4 (1.9–2.9)		1.4 (1.1–1.8)		1.8 (1.5–2.1)		1.9 (1.6–2.3)		2.2 (1.9–2.7)	
A_2_	1.6 (1.3–2.0)		1.2 (1.0–1.5)		1.5 (1.2–1.8)		1.3 (1.1–1.6)		1.7 (1.4–2.0)	

*Note*: The reference group for each analysis is children without history of parental schizophrenia spectrum disorder; *n* = number of developmentally vulnerable children with maltreatment exposure; OR, odds ratio; CI, confidence intervals; U, unadjusted; A_1_, adjusted for age, sex and socio-economic status; A_2_, adjusted for age, sex, socio-economic status and any maltreatment.

### Association of maltreatment and social-emotional functioning stratified by parental SSDs

Table 5 presents the unadjusted and adjusted associations between exposure to any maltreatment and social-emotional functioning stratified by parental SSD. In the context of having a parent with a SSD, which has an independent effect on social-emotional functioning (see Table 4), the effect of maltreatment was medium-sized for aggressive behaviour and hyperactivity/inattention, and small for poor social competency and anxious/fearful behaviour. No significant association was found for poor prosocial/helping behaviour. However, for children with no parental SSD, the effect of maltreatment was *larger* than those with parental SSD across all subdomains, particularly in poor social competency. There was little attenuation of the associations after adjusting for age, sex and SES.

**Table 5.** Associations between any maltreatment and socio-emotional functioning, stratified by history of parental schizophrenia spectrum disorder (SSD)

	Poor social competency		Poor prosocial/helping behaviour		Anxious/fearful behaviour		Aggressive behaviour		Hyperactivity/inattention	
	OR (95% CI)	*n*	OR (95% CI)	*n*	OR (95% CI)	*n*	OR (95% CI)	*N*	OR (95% CI)	*n*
*Parental SSD history*										
		39		28		53		56		75
U	1.6 (1.0–2.4)		1.2 (0.8–2.0)		1.5 (1.0–2.2)		2.4 (1.6–3.5)		2.3 (1.6–3.3)	
A	1.7 (1.1–2.6)		1.3 (0.8–2.1)		1.5 (1.1–2.3)		2.5 (1.7–3.6)		2.4 (1.7–3.5)	
*No parental SSD history*										
		285		217		327		387		423
U	3.5 (3.1–4.0)		1.8 (1.6–2.1)		1.9 (1.7–2.2)		3.3 (2.9–3.7)		2.8 (2.5–3.1)	
A	3.3 (2.9–3.8)		1.8 (1.5–2.1)		1.9 (1.7–2.1)		3.2 (2.9–3.6)		2.8 (2.5–3.2)	

*Note*: The reference group for each analysis is children experiencing no maltreatment; *n* = number of developmentally vulnerable children with maltreatment exposure; OR, odds ratio; CI, confidence intervals; U, unadjusted; A , adjusted for age, sex and socio-economic status.

## Discussion

### Associations of maltreatment and parental SSD with early social-emotional functioning

This study in a large population cohort demonstrates that exposure to early life maltreatment has adverse medium-sized effects on social competency, aggressive behaviour, and hyperactivity/inattention, and adverse small-sized effects on prosocial/helping and anxious/fearful behaviour in early childhood (age ~5 years) after adjusting for age, sex, SES and parental SSD. These results are consistent with previous research in 5–7 year-olds that did not consider parental SSDs (Anthonysamy & Zimmer-Gembeck, 2007; Milot *et al*. 2010), and with one study of older children (aged ~10 years) that showed no significant effects of parental schizophrenia on relationships between early life trauma and childhood social-emotional functioning (Bergman & Walker, 1995). Dose-dependent effects of multiple types of maltreatment were consistent with previous reports (Trickett & McBride-Chang, 1995; Lau *et al*. 2005; Vachon *et al*. 2015), and the specific types of maltreatment (physical, emotional and sexual maltreatment, and neglect) were each significantly related to a variety of social-emotional dysfunction in line with previous findings (Salzinger *et al*. 1993; Prino & Peyrot, 1994; Hildyard & Wolfe, 2002; Tyler, 2002; Schneider *et al*. 2005; Shaffer *et al*. 2009). These findings imply that exposure to greater diversity of maltreatments has a cumulative effect on social-emotional dysfunction in early childhood, but that these effects are not substantially moderated by parental SSD exposure.

Associations between maltreatment and social-emotional functioning reduced minimally after adjusting for parental SSD, and there was a greater reduction in the effect sizes of association between parental SSD and social-emotional functioning after adjusting for any maltreatment. This indicates the relatively low independent impact of parental SSD on social-emotional functioning at age 5 years in the context of childhood maltreatment exposure. The analyses stratified by parental SSD indicated relatively larger effects of maltreatment on social-emotional functioning in the group of children without parental SSD, relative to the smaller effects of maltreatment in children with parental SSD. The additional impact of early life maltreatment on childhood social-emotional functioning thus appears to be limited in children with parental history of SSD; this may reflect a tendency for children with parental history of SSD to be reported to child protection services more promptly, or at lower maltreatment thresholds, due to the parents’ increased visibility to health and other services. We were, however, underpowered to formally test for statistical interaction between parental SSD and maltreatment.

### Potential underlying mechanisms and clinical implications

Sustained exposure to stress has severe and long-term effects on brain function, including dopaminergic hyperactivity in the mesocorticolimbic system and dysregulation of the hypothalamic–pituitary–adrenal axis (Sandi & Haller, 2015). These systems are sensitised in children exposed to maltreatment (Heim & Nemeroff, 2002), in people with schizophrenia (Brunelin *et al*. 2013; Girshkin *et al*. 2014) and in first-degree relatives of people with schizophrenia (Brunelin *et al*. 2013). The extended neurodevelopmental hypothesis of schizophrenia suggests cumulative effects of stress on dopamine release and hypothalamic-pituitary-axis function that can lead to misattribution of salience to neutral stimuli (and/or impaired cognitive capacity), causing further stress and dysregulated systems, which increase the likelihood of developing schizophrenia (Morgan *et al*. 2010; Howes & Murray, 2014). The findings reinforce the importance of early identification of maltreated children and active casework by child protection agencies.

The underlying pathological processes, and the behavioural problems associated with them, may be halted or reversed with early intervention. Such interventions might include treatments targeted to maltreated children, as well as universal programs that support social-emotional learning for all children. For example, meta-analysis of studies assessing Trauma-Focused Cognitive Behavioural Therapy shows behavioural improvements in 3–18 year-old children who were exposed to maltreatment, although regular follow-up therapy may be required to achieve optimal and enduring improvements (Cary & McMillen, 2012); while meta-analysis of studies assessing school-based social and emotional competence programs indicate improved social and emotional skills, attitudes, behaviour and academic performance for children irrespective of maltreatment history (Durlak *et al*. 2011). Other interventions and policy developments implemented by government departments with responsibility for child protection services have successfully reduced maltreatment exposure [e.g., Brighter Futures Program (New South Wales Government, 2014); Positive Parenting Program (Prinz *et al*. 2009)].

### Strengths and limitations

The use of linked population data has strengths and limitations. The generalisability of our findings to other populations are supported by the use of a large, representative sample that was characterised by rates of maltreatment and parental SSDs that align with relevant recent national and international estimates. Data were collected independently of any specific hypothesis, and were not subject to participant selection or attrition (Mann, 2003). Child maltreatment data and parental SSD data were collected prospectively and independently of teacher-reports of social-emotional functioning. However, the research data were collected primarily for administrative purposes, potentially limiting the depth and accuracy of the information. Use of these administrative records will tend to most comprehensively characterise the severe end of the maltreatment and parental SSD spectrum of cases in contact with these services and miss mild cases that do not present to such services. An unknown number of maltreated children not reported to, or investigated by, child protection services and of parents with SSD not in contact with health services during the period of available data will have been misclassified as unexposed, resulting in false negatives and underestimation of the effect magnitudes. Teachers completing the AEDC may not have been blind to the maltreatment or parental SSD status of the child. We were unable to access AEDC data on 3129 children with special needs, who may present increased social-emotional vulnerability.

Another source of confounding included consideration of only parental SSD history and not SSDs in relatives beyond immediate parents. The results are also constrained by the proxy nature of some of the variables. The analyses of each maltreatment type did not exclude children that had reports of other maltreatment forms; thus, the associations reported for each type will include an unknown contribution of other forms of maltreatment. Severity of maltreatment was not assessed from the child's point of view, and not corroborated by hospital or case worker reports. Other measures of severity, for example, age at first report to child protection services, and frequency or duration of exposure, have proven useful in predicting outcome in early childhood (Tarren-Sweeney, 2008). We were unable to accurately calculate frequency of reports or duration of exposure, as one instance of maltreatment could be reported by several sources (e.g., police, family, friends, school personnel, or neighbours) resulting in multiple reports. The SEIFA measure of SES is based on a regional indication of socio-economic position and does not contain a family level indicator. Finally, we do not know if social-emotional dysfunction preceded maltreatment, so any causal relationships could not be established.

### Conclusion

Exposure to maltreatment was associated with early childhood social-emotional dysfunction, with little change to the medium-sized effects after adjusting for parental SSD and demographic covariates. Greater effects of maltreatment on social-emotional functioning were evident in children without a parental history of SSD, and the effects of parental SSD on social-emotional functioning after adjusting for maltreatment were small, suggesting greater impact of exposure to maltreatment on social-emotional functioning than parental SSDs. The influence of parental SSDs on later outcomes of maltreated children may become more apparent during follow-up of these children into adolescence and young adulthood when overt symptoms of SSD are likely to emerge. Early intervention to strengthen childhood social-emotional functioning might mitigate the impact of maltreatment, and potentially avert future psychopathology.

## Supplementary material

For supplementary material accompanying this paper visit http://dx.doi.org/10.1017/S204579601600055X. click here to view supplementary material

## Availability of data and materials

Due to ethical and data custodian requirements, data utilised in the NSW Child Development Study is not available for sharing. The research team is open to potential research collaborations with other scientists, with the proviso that analysis of linked data is currently authorised to occur at only one location, owing to ethical considerations in relation to relevant privacy legislation. In the first instance, researchers interested in potential collaboration should contact Vaughan Carr (v.carr@unsw.edu.au) with their expression of interest.
