# Supplementary material for: Effects of maltreatment and parental schizophrenia spectrum disorders on early childhood social-emotional functioning: a population record linkage study
Source: Epidemiol Psychiatr Sci. 2016 Aug 4;26(6):612–23. doi: 10.1017/S204579601600055X (PMC5729847; doi:10.1017/S204579601600055X)
Supplement: Supplementary file 1 [file S204579601600055Xsup001.docx]

**Supplementary Material**

As some previous research has identified differential effects of maltreatment on social-emotional functioning in young females and males^1^, this supplementary material summarises the results of exploratory analyses conducted separately by sex.

*Sample characteristics by sex*: The study sample of 69,116 children included 34,185 females (49.5%). Child protection reports dated prior to the AEDC assessment were recorded for 1,048 females and 993 males. The female and male prevalence rates for each type of maltreatment (at least one report) were as follows: physical - 222 females, 238 males; emotional - 563 females, 559 males; sexual - 145 females, 78 males; and neglect - 359 females, 362 males. Regarding diversity of maltreatment, 841 females and 785 males had a report of one type of maltreatment only, and 207 females and 208 males had reports of two or more types of maltreatment. A parental history of schizophrenia spectrum disorder (SSD) was present for 410 females and 425 males, and 120 females and 117 males had both a child protection report and at least one parent with a SSD.

*Associations by sex:* Analyses reported within the supplementary tables were conducted as described in the manuscript, with the exception that sex was removed as a covariate and analyses were instead conducted separately for females and males. Overall, significant associations were apparent for both sexes, and the confidence intervals for males and females overlapped in most cases. Similarities and differences in the pattern and magnitude of effects observed for females and males are described following.

Supplementary Table 1 summarises the results of unadjusted and adjusted analyses between any maltreatment exposure and social-emotional outcomes for females and males. Effects of medium magnitude were observed in fully adjusted analyses between any maltreatment and poor social competency, aggressive behaviour, and hyperactivity/inattention for both sexes, and for anxious/fearful behaviour in females only (where the association in males was small in magnitude). Small effects characterised the association between any maltreatment and poor prosocial/helping behaviour in females and males.

Supplementary Table 2 indicates the associations by sex between maltreatment type and social-emotional functioning. The effect sizes obtained for females were larger in magnitude across all subdomains than those obtained for males, except for sexual maltreatment, where the pattern was in the opposite direction. For females, physical maltreatment had the greatest impact of any maltreatment type on poor social competency, anxious/fearful behaviour, aggressive behaviour, and hyperactive-inattentive behaviour, while neglect had the greatest impact on poor prosocial/helping behaviour. For males, physical maltreatment had the greatest impact of any type of maltreatment on poor social competency and aggressive behaviour, while sexual maltreatment had the greatest impact on poor prosocial/helping behaviour, anxious/fearful behaviour, and hyperactive-inattentive behaviour.

Supplementary Table 3 indicates dose-dependent associations for both sexes between diversity of maltreatment and social-emotional functioning. For the poor social competency, aggressive behaviour, and hyperactivity/inattention subdomains, effect sizes increased from medium to large for females and from medium to medium-to-large for males. For anxious/fearful behaviour, the effect sizes increased from small to medium for females; a limited dose-dependent association was apparent for males, with the effect size remaining of small magnitude even for boys who had experienced two or more types of maltreatment. For poor prosocial/helping behaviour, a dose-dependent association was apparent only for males, though the effect sizes remained small.

Overall, while the confidence intervals for males and females overlapped in most cases, effect sizes were slightly greater in magnitude for females (relative to those for males) for all AEDC subdomains, and for all types of maltreatment except sexual maltreatment. This might reflect the timing of maltreatment exposure; previous research has indicated that females exposed to maltreatment prior to age four years show increasing internalizing and externalizing behaviours over time compared to females not exposed, while males show the opposite effect over time^1^. While further analysis in our sample might usefully delineate maltreatment exposure during infancy, toddlerhood, and pre-schoolers, we did not conduct this analysis due to unknown delays between maltreatment exposure and filing of child protection reports^2^. With regard to the effects of specific types of maltreatment, physical maltreatment had the greatest impact on social-emotional functioning in both males and females, with sexual maltreatment also having strong impact on social-emotional functioning in males. Sexual maltreatment has been reported previously to have greater impact on social-emotional functioning in males than females^3-5^. This may be explained by sexual maltreatment involving greater force and physical harm in males^6, 7^, particularly when the perpetrator is male, which may manifest feelings of humiliation, guilt, shame, and confusion^8^. Further data linkages planned in this cohort during middle childhood, adolescence, and adulthood^9^ will provide capacity to examine later life impacts of early maltreatment exposure on social-emotional functioning in females and males.

Table 1: Associations between exposure to any maltreatment and socio-emotional functioning reported separately for females and males

|  | **Poor social competency** | | **Poor prosocial / helping behaviour** | | **Anxious / fearful behaviour** | | **Aggressive behaviour** | | **Hyperactivity / inattention** | |
| --- | --- | --- | --- | --- | --- | --- | --- | --- | --- | --- |
|  | OR (95%CI) | n | OR (95%CI) | n | OR (95%CI) | n | OR (95%CI) | n | OR (95%CI) | n |
| ***Females*** | | | | | | | | | | |
|  |  | 132 |  | 81 |  | 190 |  | 139 |  | 174 |
| U: | 3.9 (3.2-4.7) |  | 2.1 (1.7-2.7) |  | 2.2 (1.9-2.6) |  | 3.6 (3.0-4.4) |  | 4.0 (3.3-4.7) |  |
| A_1_: | 3.5 (2.9-4.2) |  | 1.9 (1.5-2.4) |  | 2.1 (1.8-2.5) |  | 3.4 (2.8-4.2) |  | 3.7 (3.1-4.4) |  |
| A_2_: | 3.4 (2.8-4.1) |  | 1.9 (1.5-2.5) |  | 2.0 (1.7-2.4) |  | 3.2 (2.7-3.9) |  | 3.4 (2.9-4.1) |  |
| ***Males*** | | | | | | | | | | |
|  |  | 192 |  | 164 |  | 190 |  | 304 |  | 324 |
| U: | 3.4 (2.9-4.0) |  | 1.8 (1.5-2.1) |  | 1.8 (1.6-2.2) |  | 3.4 (3.0-3.9) |  | 2.8 (2.4-3.2) |  |
| A_1_: | 3.2 (2.7-3.7) |  | 1.7 (1.4-2.0) |  | 1.7 (1.5-2.1) |  | 3.2 (2.7-3.6) |  | 2.6 (2.3-3.0) |  |
| A_2_: | 2.9 (2.5-3.5) |  | 1.6 (1.4-1.9) |  | 1.7 (1.4-2.0) |  | 3.1 (2.7-3.6) |  | 2.5 (2.1-2.8) |  |

Note: The reference group for each analysis is children experiencing no maltreatment; n = number of developmentally vulnerable children with maltreatment exposure; OR = odds ratio; CI = confidence intervals; U = unadjusted; A_1_ = adjusted for age and socio-economic status; A_2_ = adjusted for age, socio-economic status, and parental SSD

Table 2: Associations between exposure to each type of maltreatment and socio-emotional functioning reported separately for females and males

|  | **Poor social competency** | | **Poor prosocial / helping behaviour** | | **Anxious / fearful behaviour** | | **Aggressive behaviour** | | **Hyperactivity / inattention** | |
| --- | --- | --- | --- | --- | --- | --- | --- | --- | --- | --- |
|  | OR (95%CI) | n | OR (95%CI) | n | OR (95%CI) | n | OR (95%CI) | n | OR (95%CI) | n |
| ***Females*** | | | | | | | | | | |
| **Physical maltreatment** | | 37 |  | 16 |  | 49 |  | 41 |  | 45 |
| U: | 5.4 (3.8-7.7) |  | 2.0 (1.2-3.3) |  | 2.9 (2.1-3.9) |  | 5.4 (3.9-7.7) |  | 5.1 (3.7-7.1) |  |
| A_1_: | 4.8 (3.3-6.9) |  | 1.8 (1.1-3.0) |  | 2.7 (2.0-3.7) |  | 5.1 (3.6-7.2) |  | 4.7 (3.4-6.6) |  |
| A_2_: | 4.6 (3.2-6.6) |  | 1.7 (1.0-2.9) |  | 2.6 (1.9-3.5) |  | 4.8 (3.4-6.9) |  | 4.4 (3.2-6.2) |  |
| **Emotional maltreatment** | | 82 |  | 34 |  | 116 |  | 81 |  | 103 |
| U: | 4.6 (3.6-5.9) |  | 1.6 (1.2-2.3) |  | 2.6 (2.1-3.2) |  | 4.0 (3.2-5.1) |  | 4.5 (3.6-5.6) |  |
| A_1_: | 4.1 (3.2-5.2) |  | 1.5 (1.0-2.1) |  | 2.5 (2.0-3.1) |  | 3.8 (3.0-4.8) |  | 4.1 (3.1-5.1) |  |
| A_2_: | 3.8 (3.0-4.9) |  | 1.5 (1.0-2.1) |  | 2.3 (1.8-2.8) |  | 3.5 (2.7-4.5) |  | 3.7 (2.9-4.7) |  |
| **Sexual maltreatment** | | 16 |  | 12 |  | 29 |  | 16 |  | 16 |
| U: | 3.3 (2.0-5.6) |  | 2.2 (1.2-4.0) |  | 2.5 (1.6-3.7) |  | 2.9 (1.7-4.9) |  | 2.4 (1.4-4.1) |  |
| A_1_: | 3.1 (1.8-5.2) |  | 2.1 (1.1-3.7) |  | 2.4 (1.6-3.6) |  | 2.8 (1.7-4.7) |  | 2.3 (1.4-3.9) |  |
| A_2_: | 3.0 (1.8-5.2) |  | 2.0 (1.1-3.7) |  | 2.4 (1.6-3.5) |  | 2.8 (1.6-4.6) |  | 2.3 (1.3-3.8) |  |
| **Neglect** | | 44 |  | 36 |  | 56 |  | 56 |  | 69 |
| U: | 3.8 (2.7-5.2) |  | 2.8 (2.0-4.0) |  | 1.8 (1.4-2.4) |  | 4.4 (3.3-5.8) |  | 4.7 (3.6-6.1) |  |
| A_1_: | 3.4 (2.4-4.6) |  | 2.5 (1.8-3.6) |  | 1.7 (1.3-2.3) |  | 4.1 (3.1-5.5) |  | 4.4 (3.3-5.7) |  |
| A_2_: | 3.3 (2.3-4.5) |  | 2.5 (1.7-3.6) |  | 1.6 (1.2-2.2) |  | 3.8 (2.9-5.2) |  | 4.1 (3.1-5.4) |  |
| ***Males*** | | | | | | | | | | |
| **Physical maltreatment** | | 52 |  | 32 |  | 43 |  | 85 |  | 82 |
| U: | 4.0 (3.0-5.5) |  | 1.4 (0.9-2.0) |  | 1.7 (1.2-2.4) |  | 4.4 (3.3-5.7) |  | 3.0 (2.3-4.0) |  |
| A_1_: | 3.8 (2.8-5.2) |  | 1.3 (0.9-1.9) |  | 1.6 (1.2-2.3) |  | 4.1 (3.1-5.3) |  | 3.0 (2.2-3.8) |  |
| A_2_: | 3.5 (2.5-4.8) |  | 1.3 (0.9-1.9) |  | 1.6 (1.1-2.2) |  | 4.0 (3.0-5.2) |  | 2.8 (2.1-3.6) |  |
| **Emotional maltreatment** | | 116 |  | 98 |  | 104 |  | 175 |  | 190 |
| U: | 3.7 (3.0-4.6) |  | 1.9 (1.5-2.4) |  | 1.8 (1.4-2.2) |  | 3.5 (2.9-4.2) |  | 2.9 (2.4-3.5) |  |
| A_1_: | 3.4 (2.7-4.2) |  | 1.8 (1.4-2.2) |  | 1.7 (1.3-2.1) |  | 3.2 (2.7-3.9) |  | 2.7 (2.3-3.2) |  |
| A_2_: | 3.1 (2.5-3.8) |  | 1.7 (1.4-2.1) |  | 1.6 (1.3-2.0) |  | 3.1 (2.6-3.8) |  | 2.5 (2.1-3.1) |  |
| **Sexual maltreatment** | | 16 |  | 15 |  | 20 |  | 25 |  | 30 |
| U: | 3.7 (2.1-6.4) |  | 2.1 (1.2-3.7) |  | 2.7 (1.6-4.5) |  | 3.6 (2.3-5.9) |  | 3.6 (2.2-5.6) |  |
| A_1_: | 3.6 (2.1-6.2) |  | 2.1 (1.1-3.5) |  | 2.6 (1.6-4.3) |  | 3.5 (2.2-5.6) |  | 3.5 (2.2-5.6) |  |
| A_2_: | 3.2 (1.9-5.7) |  | 2.0 (1.1-3.5) |  | 2.5 (1.5-4.2) |  | 3.4 (2.1-5.5) |  | 3.3 (2.1-5.3) |  |
| **Neglect** | | 77 |  | 64 |  | 76 |  | 119 |  | 128 |
| U: | 3.9 (3.0-5.0) |  | 1.9 (1.5-2.6) |  | 2.1 (1.6-2.7) |  | 3.8 (3.1-4.8) |  | 3.2 (2.5-3.9) |  |
| A_1_: | 3.6 (2.8-4.6) |  | 1.8 (1.4-2.4) |  | 2.0 (1.5-2.6) |  | 3.5 (2.8-4.4) |  | 3.0 (2.4-3.7) |  |
| A_2_: | 3.3 (2.5-4.2) |  | 1.8 (1.4-2.4) |  | 1.9 (1.5-2.5) |  | 3.5 (2.8-4.3) |  | 2.8 (2.2-3.5) |  |

Note: The reference group for each analysis is children experiencing no maltreatment; n = number of developmentally vulnerable children with maltreatment exposure; OR = odds ratio; CI = confidence intervals; U = unadjusted; A_1_ = adjusted for age and socio-economic status; A_2_ = adjusted for age, socio-economic status, and parental SSD

Table 3: Associations between exposure to diversity of maltreatment and socio-emotional functioning reported separately for females and males

|  | | **Poor social competency** | | **Poor prosocial / helping behaviour** | | **Anxious / fearful behaviour** | | **Aggressive behaviour** | | **Hyperactivity / inattention** | |
| --- | --- | --- | --- | --- | --- | --- | --- | --- | --- | --- | --- |
|  | | OR (95%CI) | n | OR (95%CI) | n | OR (95%CI) | n | OR (95%CI) | n | OR (95%CI) | n |
| ***Females*** | | | | | | | | | | | |
| **1 type** | | | 92 |  | 65 |  | 138 |  | 93 |  | 122 |
|  | U: | 3.3 (2.6-4.1) |  | 2.1 (1.6-2.7) |  | 2.0 (1.6-2.4) |  | 3.0 (2.4-3.7) |  | 3.4 (2.8-4.1) |  |
|  | A_1_: | 3.0 (2.4-3.7) |  | 1.9 (1.5-2.5) |  | 1.9 (1.6-2.3) |  | 2.8 (2.2-3.5) |  | 3.2 (2.6-3.9) |  |
|  | A_2_: | 2.9 (2.3-3.6) |  | 1.9 (1.5-2.5) |  | 1.8 (1.5-2.2) |  | 2.7 (2.1-3.3) |  | 2.9 (2.4-3.6) |  |
| **≥2 types** | |  | 40 |  | 16 |  | 52 |  | 46 |  | 52 |
|  | U: | 6.4 (4.5-9.2) |  | 2.1 (1.3-3.5) |  | 3.3 (2.4-4.6) |  | 6.8 (4.8-9.4) |  | 6.6 (4.8-9.1) |  |
|  | A_1_: | 5.7 (4.0-8.1) |  | 1.9 (1.1-3.2) |  | 3.2 (2.3-4.3) |  | 6.3 (4.5-8.8) |  | 6.1 (4.4-8.5) |  |
|  | A_2_: | 5.5 (3.8-7.9) |  | 1.9 (1.1-3.2) |  | 3.0 (2.2-4.1) |  | 5.9 (4.2-8.3) |  | 5.7 (4.1-7.9) |  |
| ***Males*** | | | | | | | | | | | |
| **1 type** | | | 133 |  | 125 |  | 146 |  | 219 |  | 233 |
|  | U: | 2.9 (2.4-3.5) |  | 1.7 (1.4-2.1) |  | 1.8 (1.5-2.1) |  | 3.0 (2.5-3.5) |  | 2.4 (2.0-2.8) |  |
|  | A_1_: | 2.7 (2.2-3.3) |  | 1.6 (1.3-2.0) |  | 1.7 (1.4-2.0) |  | 2.8 (2.4-3.3) |  | 2.3 (1.9-2.6) |  |
|  | A_2_: | 2.5 (2.1-3.1) |  | 1.6 (1.3-1.9) |  | 1.6 (1.3-2.0) |  | 2.7 (2.3-3.2) |  | 2.2 (1.8-2.5) |  |
| **≥2 types** | |  | 59 |  | 39 |  | 44 |  | 85 |  | 91 |
|  | U: | 5.7 (4.2-7.7) |  | 2.1 (1.5-2.9) |  | 2.1 (1.5-2.9) |  | 5.5 (4.1-7.2) |  | 4.5 (3.4-6.0) |  |
|  | A_1_: | 5.1 (3.8-7.0) |  | 1.9 (1.3-2.7) |  | 2.0 (1.4-2.7) |  | 4.9 (3.7-6.6) |  | 4.2 (3.1-5.5) |  |
|  | A_2_: | 4.7 (3.4-6.4) |  | 1.8 (1.3-2.6) |  | 1.9 (1.4-2.0) |  | 4.8 (3.6-6.4) |  | 3.9 (2.9-5.2) |  |

Note: The reference group for each analysis is children experiencing no maltreatment; n = number of developmentally vulnerable children with maltreatment exposure; OR = odds ratio; CI = confidence intervals; U = unadjusted; A_1_ = adjusted for age and socio-economic status; A_2_ = adjusted for age, socio-economic status, and parental SSD

**Supplementary material references**

**1.** Godinet MT, Li F, Berg T. Early childhood maltreatment and trajectories of behavioral problems: exploring gender and racial differences. *Child Abuse Negl* 2014;38(3):544-556.

**2.** Lau AS, Leeb RT, English D, Graham JC, Briggs EC, Brody KE, Marshall JM. What’s in a name? A comparison of methods for classifying predominant type of maltreatment. *Child Abuse Negl* 2005;29 533-551.

**3.** Garnefski N, Diekstra RF. Child sexual abuse and emotional and behavioral problems in adolescence: gender differences. *J Am Acad Child Adolesc Psychiatry* 1997;36(3):323-329.

**4.** Coohey C. Gender differences in internalizing problems among sexually abused early adolescents. *Child Abuse Negl* 2010;34(11):856-862.

**5.** Estes LS, Tidwell R. Sexually abused children's behaviours: impact of gender and mother's experience of intra- and extra-familial sexual abuse. *Fam Pract* 2002;19(1):36-44.

**6.** Ellerstein NS, Canavan JW. Sexual Abuse of Boys. *Am J Dis Child* 1980;134(3):255-257.

**7.** Spataro J, Moss SA, Wells DL. Child Sexual Abuse: A Reality for Both Sexes. *Aust Psychol* 2001;36(3):177-183.

**8.** Cashmore J, Shackel R. Gender Differences in the Context and Consequences of Child Sexual Abuse. *CICrimJust* 2014;24(1):75-104.

**9.** Carr VJ, Harris F, Raudino A, et al. Cohort Profile: The New South Wales Child Development Study (NSW-CDS) – An Australian multiagency, multi-generational, longitudinal record linkage study. *BMJ Open* Accepted.
